# In Vitro Analysis of Enamel Patterns Across Three Species Using Stereomicroscopy

**DOI:** 10.7759/cureus.59488

**Published:** 2024-05-01

**Authors:** Akshai Senthilkumar, Ramya Ramadoss, Karthikeyan Ramalingam, Abirami Arthanari

**Affiliations:** 1 Forensic Odontology, Saveetha Dental College and Hospitals, Saveetha Institute of Medical and Technical Sciences, Saveetha University, Chennai, IND; 2 Oral Pathology and Oral Biology, Saveetha Dental College and Hospitals, Saveetha Institute of Medical and Technical Sciences, Saveetha University, Chennai, IND; 3 Oral Pathology and Microbiology, Saveetha Dental College and Hospitals, Saveetha Institute of Medical and Technical Sciences, Saveetha University, Chennai, IND

**Keywords:** research dentistry, forensic odontology, enamel prisms, forensic patterns, enamel rod-end patterns, enamel patterns, tooth prints, perikymata patterns, enamel prints, ameloglyphics

## Abstract

Background

Dental enamel, the outermost layer of the tooth, stands as a unique and remarkable tissue that plays a crucial role in safeguarding teeth against various external factors. The examination and analysis of enamel rod end patterns on tooth surfaces, referred to as ameloglyphics, offer a promising avenue for dental identification and forensic investigations in forensic medicine, thereby enhancing the precision and reliability of forensic analyses. This paper aims to evaluate and compare the ameloglyphics of different species under a stereomicroscope. The species examined in this study include the beaver (genus *Castor*), fox (genus *Vulpes*), and human (*Homo sapiens*).

Methods

Teeth samples from each species (n = 3) were gathered from the tooth repository and examined under a stereomicroscope at various magnifications, both with and without graphite staining. Photographs were captured, and the enamel patterns were meticulously evaluated. Through the utilization of plot profiles, the enamel patterns of the different species were compared, and any discernible differences between them were carefully noted.

Results

The plot profiles of the three species were analyzed, revealing distinct characteristics. Specifically, it was observed that the plots of the genus *Castor* teeth and *Homo sapiens* teeth exhibited dispersed arrangements, whereas the plot profile of the genus *Vulpes* displayed a closely arranged pattern.

Conclusions

Within the confines of the current investigation, it can be inferred that every mammal exhibits a distinct and exclusive arrangement of enamel rods. Hence, the task of identifying a deceased individual through dental imprints may pose difficulties if the structural characteristics are not thoroughly comprehended.

## Introduction

Enamel, acknowledged as the hardest and strongest component within mammalian teeth, derives its functionality from its complex internal framework. Upon closer examination at higher magnifications, the intricate internal structures of enamel become readily discernible, revealing the remarkable intricacies of its composition and organization [1]. When observed at a macroscopic scale upon the tooth's surface, the arrangement of enamel rods forms a distinctive incremental, wave-like pattern known as perikymata. Throughout history, alterations to these structures have primarily occurred due to various factors such as mechanical abrasion, chemical erosion, or gradual attrition, thereby underscoring the multifaceted nature of their susceptibility to modification and wear [2]. Depending on factors, such as age, oral hygiene practices, and the alignment of teeth, the loss of perikymata could serve as notable indicators in the field of forensic science, offering valuable insights into an individual's personal history and habits [3].

Perikymata, situated externally, and striae of Retzius, internally, are indicative of the developmental features present on the lateral and cervical surfaces of tooth enamel. As perikymata do not reach the cuspal surface of the enamel, it is essential to carefully examine a tooth, whether through natural wear or sectioning, to identify perikymata patterns accurately. This assessment is integral for comprehending the developmental timeline and structural attributes of tooth enamel [4]. Differences in the shape and configuration of particular features have proven invaluable in discerning between species and constructing phylogenetic relationships. Notably, the enamel prism structures exhibit distinct variations: herbivores typically display elliptical or slender formations, carnivores tend to exhibit round structures, while omnivores showcase patterns resembling either a circular arc or an elliptical configuration. These distinctive characteristics serve as significant indicators for taxonomic classification and evolutionary analysis [5].

Due to its remarkably high mineral content, dentition emerges as an exceptionally abundant and enduring resource within the fossil record, persisting throughout various geological epochs and providing a rich source of information for paleontologists and evolutionary biologists alike [2]. Crocodiles, along with most other reptiles, have a dental phenomenon called polyphyodonty. This means that they continuously replace their teeth throughout their lives. Unlike mammals, whose teeth only grow and are replaced twice (deciduous teeth and permanent teeth), crocodiles and other reptiles continually grow new teeth to replace ones that are worn down or lost due to injury or other factors. This adaptation allows them to maintain functional teeth throughout their lifespan, ensuring effective feeding and survival. In contrast, mammals, including humans, exhibit diphyodonty, where they only have two sets of teeth: the deciduous teeth, which are replaced by permanent teeth during development, and the permanent teeth, which are retained for the rest of their lives [6]. This significant contrast in dental structure highlights the evolutionary divergence between reptiles, like crocodiles and mammals. In *Psarolepis romeri*, an ancient species dating back approximately 400 million years, the majority of teeth were predominantly composed of dentine. This stands in contrast to ancient fish, which possessed enamel-like material on their scales, with true enamel evolving gradually over time. Interestingly, the thickness of enamel seems to correlate with an animal's longevity, with longer-living mammals, including humans, typically exhibiting greater enamel thickness. This suggests an evolutionary adaptation related to the durability and lifespan of the species [7].

When examined at a microscopic scale, enamel prisms exhibit a distinctive arrangement wherein they form groups running in different directions from one another. This organization gives rise to diverse patterns of enamel rod endings [8]. The examination and analysis of enamel rod end patterns on tooth surfaces are referred to as ameloglyphics [9]. Ameloglyphics, a specialized field within forensic dentistry, has emerged as a crucial tool, especially in the aftermath of catastrophic events, such as natural disasters, where mass fatalities occur. Through the meticulous analysis of enamel rod end patterns on tooth surfaces, this discipline provides valuable insights into the identification process, aiding forensic experts in accurately identifying victims and providing closure to their families [10]. The study of dental hard tissues, notably enamel, holds immense value for post-mortem identification, with dental evidence often establishing compelling associations. Enamel, characterized by its exceptional resilience, may even withstand deterioration post-mortem. Crucially, the distinct enamel rod end pattern unique to each individual serves as compelling evidence within the realm of forensic odontology, facilitating accurate identification processes [9].

The study of ameloglyphics, examining enamel prints left by animals, offers invaluable insights into behavior and ecological dynamics. While beavers' distinctive prints help track their behavior and role in forest ecosystems, fox enamel prints similarly contribute to wildlife management and conservation. The varying rate of enamel secretion, influenced by factors like metabolic rate and diet, impacts dental health and ecological adaptations across species. Despite challenges, tooth repositories and specialized facilities enable research on beaver enamel prints, advancing ecological understanding. In India, where beavers are absent, conducting in vitro studies on their enamel prints presents difficulties due to the lack of specimens and specialized facilities. Nevertheless, researchers have overcome these obstacles by utilizing tooth repositories and collaborating internationally, thus enhancing understanding of this ecological phenomenon despite the logistical constraints.

Numerous studies have extensively examined the microstructure of tooth enamel, spanning from dinosaurs to humans, shedding light on the evolutionary and biological insights garnered from this diverse array of organisms [2]. In a similar vein, previous research has delved into various aspects, including the micro-wear texture of mammals, dietary adaptations among different mammalian species, and comparative analyses between humans and carnivores [11,12]. However, the quantification and detailed comparison of ameloglyphics among different mammalian groups have been notably absent from prior discussions. In light of this gap in research, our study represents a pioneering effort aimed at evaluating and comparing ameloglyphics across various species using the stereomicroscope. Different species compared in this study were beavers (genus *Castor*), foxes (genus *Vulpes*), and humans (*Homo sapiens*). Beavers are herbivores, foxes are predominantly carnivores [13], and humans are categorized as omnivores.

## Materials and methods

The research was conducted in vitro at the esteemed Department of Oral Biology, with approval obtained from the Scientific Review Board at Saveetha Dental College and Hospitals, Chennai, India (approval number SDC/PhD/OPATH-2212/22/001). The extensive collection of teeth samples representing various species was carefully procured from the renowned tooth repository housed within Saveetha Dental College. To maintain the quality of the samples, a rigorous disinfection process using 10% formaldehyde was meticulously carried out, followed by careful air drying and storage in tightly sealed containers until they were ready for analysis.

**Figure 1:**
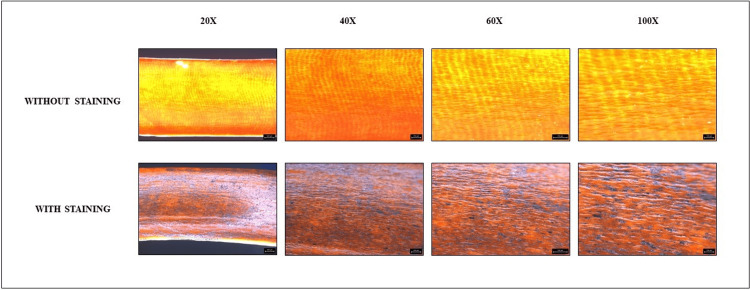
Genus Castor (beaver) teeth under a stereomicroscope at different magnifications With staining using graphite pencil.

**Figure 2:**
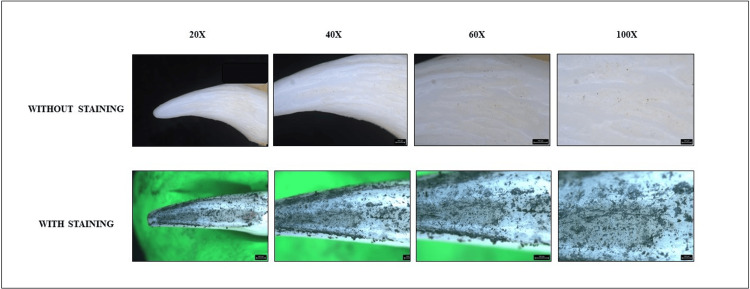
Genus Vulpes (fox) teeth under a stereomicroscope at different magnifications With staining using graphite pencil.

**Figure 3:**
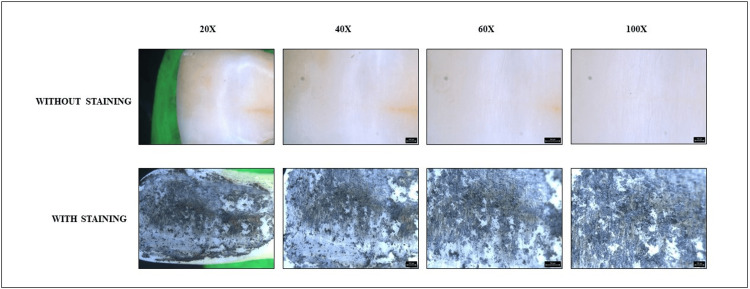
Homo sapiens (human) teeth under a stereomicroscope at different magnifications With staining using graphite pencil.

Following the preparation phase, a direct application of graphite was methodically performed on the selected teeth intended for the study. This process involved the precise application of graphite using a pencil, ensuring uniform coverage of the tooth surfaces. Subsequently, the prepared specimens were delicately mounted onto wax sheets and subjected to thorough examination (before and after staining) under the precise lens of a stereomicroscope (Leica Microsystems GmbH, Wetzlar, Germany). The examination was meticulously conducted across a spectrum of magnifications, encompassing intervals of 20×, 40×, 60×, and 100×, with assessments performed both prior to and subsequent to the application of graphite coatings, as exemplified in the visual representations provided in Figure 1, Figure 2, and Figure 3. Subsequent analysis involved the meticulous measurement of the distance between individual enamel rods and the generation of plot profiles using sophisticated software tools such as ImageJ, developed by the National Institutes of Health (public domain open source software, National Institute of Mental Health, Bethesda, Maryland, USA). The vast dataset obtained was meticulously processed and analyzed using industry-standard software such as Microsoft Excel. Particular attention was paid to any discernible discrepancies in enamel rod patterns between the graphite-stained and unstained images, with comprehensive comparisons undertaken to elucidate any significant findings. Figure 1, Figure 2, and Figure 3 represent the graphite stained and unstained images of genus *Castor*, genus *Vulpes*, and *Homo sapiens* under different magnifications, respectively.

## Results

In the control group, only images captured under 100× magnification were employed for measurements. Examination of the gathered data unveiled variations in the distance between individual enamel rods among species: for the genus *Vulpes*, it registered at 520.035 µm; for the genus *Castor*, it stood at 228.035 µm; and for *Homo sapiens*, it recorded 67.007 µm. Additionally, the area of teeth for the *Vulpes *genus is 521 µm, with a length of 520.035 µm, a mean of 200.77 µm, a minimum of 191.53 µm, and a maximum of 211.32 µm. For the *Castor *genus, the area measures 229 µm, with a length of 228.035 µm, a mean of 152.24 µm, a minimum of 144.49 µm, and a maximum of 162.38 µm. The area of teeth for *Homo sapiens* is 68 µm, with a length of 67.007 µm, a mean of 228.65 µm, a minimum of 218.84 µm, and a maximum of 237.09 µm.

Table 1 furnishes a detailed compilation of the values extracted from the ImageJ software for different species, offering a thorough overview of the findings.

**Table 1:** Values obtained from ImageJ software for different species Only the control group is under 100× magnification. *All units are expressed in µm.

Teeth	Area	Length	Mean	Min	Max
Genus *Castor*	229	228.035	152.24	144.49	162.38
Genus *Vulpes*	521	520.035	200.77	191.53	211.32
Homo sapiens	68	67.007	228.65	218.84	237.09

**Figure 4:**
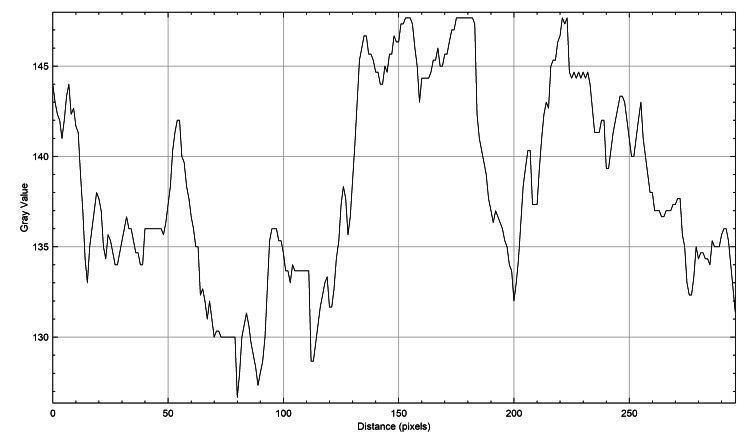
Beaver teeth plot profile The x-axis represents the distance along the line, and the y-axis is the pixel intensity.

**Figure 5:**
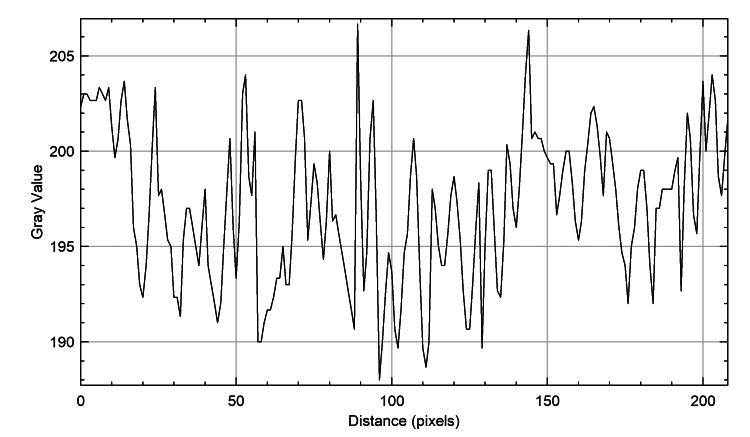
Fox teeth plot profile The x-axis represents the distance along the line, and the y-axis is the pixel intensity.

**Figure 6:**
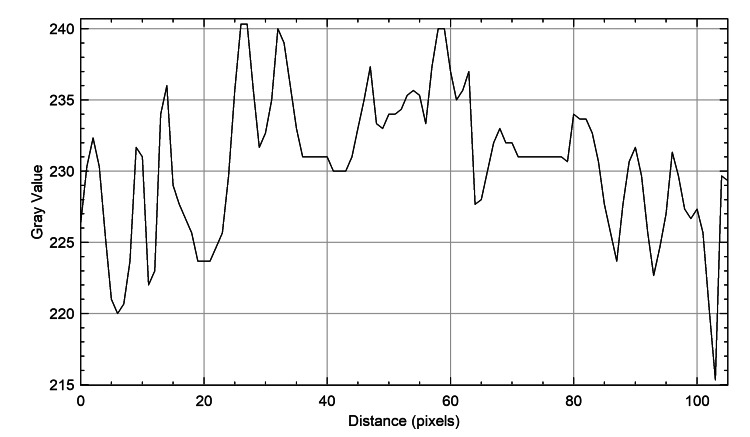
Human teeth plot profile The x-axis represents the distance along the line, and the y-axis is the pixel intensity.

The plot profiles of the three species were meticulously analyzed to discern their unique characteristics. It was observed that the plot profiles of the genus *Castor *teeth (Figure 4) and *Homo sapiens* teeth (Figure 5) displayed a dispersed arrangement, whereas the plot profile of the genus *Vulpes *(Figure 6) exhibited a closely arranged pattern. This distinction was visually represented in figures, each illustrating the plot profiles of the respective species in detail.

**Figure 7:**
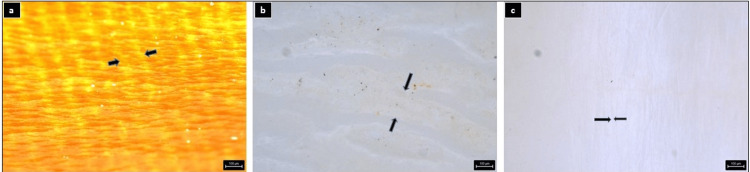
Images that were used to analyze the ameloglyphic patterns of (a) genus Castor, (b) genus Vulpes, and (c) Homo sapiens, respectively. Arrowheads indicate the distance between two enamel rods, which were used for examination.

Figure 7 shows the images that were used to analyze the ameloglyphic patterns of beavers (genus *Castor*), foxes (genus *Vulpes*), and humans (*Homo sapiens*), respectively.

Subsequently, a comparison between the stained and unstained images was conducted. Surprisingly, it was found that the graphite coating method of staining failed to effectively display the enamel rod patterns under the stereomicroscope for all the species examined. This observation underscores the need for further refinement in staining techniques to enhance their efficacy in visualizing enamel rod patterns.

## Discussion

Dentin is exposed when the protective layer of enamel rod ends is worn away by brushing, eating hard foods, and being in acidic environments at work [14,15]. It is critical to better understand how these processes affect the enamel rods' quality [16-18]. Enamel is the hardest structure with the best organization and mineralization of any other substance in the human body [19,20]. After establishing itself, enamel neither remodels nor stays in contact with ameloblasts or secretory cells. Once enamel is formed, ameloblasts deposit enamel rods in an alternating pattern, i.e., they either move or retract from the enamel surface and leave behind the prism morphology [7]. Ameloglyphics, or the study of enamel print patterns, is regarded as a very trustworthy biometric-based method for human identification [15].

The exploration of ameloglyphics delves into analyzing enamel impressions left by various creatures, offering invaluable insights into behavioral and ecological dynamics. Beavers' unique prints aid in monitoring their behavior and role in forest ecosystems, while fox enamel prints similarly contribute to wildlife management and conservation. The diverse rate of enamel secretion, influenced by factors like metabolic rate and diet, affects dental health and ecological adaptations across species. Despite logistical challenges, tooth repositories and specialized facilities facilitate research on beaver enamel prints, advancing ecological comprehension. In regions lacking beavers, like India, conducting in vitro studies on their enamel prints poses difficulties, yet researchers have overcome these obstacles by utilizing tooth repositories and international collaborations. Additionally, understanding human ameloglyphics is crucial for forensic science, anthropology, and dentistry, aiding in individual identification and assessing dental health. This exploration encompasses a study involving beavers, foxes, and humans, revealing novel distinctions and shedding light on the significance of enamel impressions. By uncovering distinct patterns in enamel secretion rates among these species, this research offers valuable contributions to wildlife management and conservation efforts, underscoring the diverse applications and potential of ameloglyphics to advance our understanding of wildlife biology and ecosystem dynamics.

Various techniques have been extensively explored by numerous authors to investigate enamel rod patterns. These methods include the acetate peel technique, cellophane tape technique, acid etching, automated biometric technique, scanning electron microscope, and stereomicroscope [16,17,21]. While each method offers its advantages, electron microscopy and stereomicroscopy stand out for their higher magnification, providing detailed views of enamel structures. Following scanning electron microscopy, the stereomicroscope emerged as the most suitable method for studying ameloglyphics. Despite the advantages of electron microscopy and stereomicroscopy, other methods hold significance due to their simplicity and lack of additional preparation requirements. Despite the requirement for antemortem records, the stereomicroscope offered reliability and precision, making it the preferred method for our research subsequent to scanning electron microscopy.

The present study undertook the quantification of enamel rod patterns observed in three distinct species, specifically the genus *Castor*, genus *Vulpes*, and *Homo sapiens*. Utilizing a stereomicroscope, these patterns were meticulously visualized at varying magnifications, both before and following the application of graphite coatings. Drawing parallels with our methodology, findings by Narayan VK et al. (2017) underscored the instrumental role of stereomicroscope in achieving a more precise identification of the mean age difference between actual and estimated age, with an impressive precision level of up to ±1.0 years [17].

Upon comparing the distance between one enamel rod to another among all species, it was observed that the values for the genus *Vulpes* were greater than those for the genus *Castor *and *Homo sapiens*. This disparity can be attributed to variations in the rate of enamel secretion across different species. Furthermore, perikymata were found to be more prominent in herbivores compared to carnivores in our study, aligning with research by Ziedonis Skobe et al. (1985), who concluded that a primary distinction between the teeth of carnivores and humans is that the growth lines of the former do not terminate at perikymata on the tooth's surface [12].

The direct method of graphite coat staining was found to be ineffective in displaying the enamel rod patterns for all the species. In contrast to our study, Dinesh Yasothkumar et al., in the year 2021, evaluated the enamel rod patterns of 27 maxillary premolars using various staining methods under a stereomicroscope. They concluded that the soak method of staining was more effective than the other two methods [18].

The plot profiles of the three species were analyzed, revealing that the plots of genus *Castor *teeth and *Homo sapiens* teeth exhibited striking similarities, whereas the plot profile of genus *Vulpes* displayed distinct differences. Despite the specific focus on species like beavers (genus *Castor*), foxes (genus *Vulpes*), and humans (*Homo sapiens*), it is important to recognize the limitations of this study. These include challenges such as small sample sizes and the direct application of graphite stains to specimens. However, despite these obstacles, the study remains groundbreaking in its assessment and comparison of ameloglyphics using a stereomicroscope with direct graphite staining. Acknowledging the narrow scope of the research, it is crucial to consider the potential limitations in generalizing the findings to other species. To address this, larger-scale studies encompassing a broader range of species are essential. Future studies will allow for a more thorough evaluation of staining methods and enhance the applicability of the results. Such comprehensive investigations will provide a deeper understanding of enamel patterns across different taxa and facilitate the development of standardized protocols for enamel analysis in forensic and ecological studies. Moreover, this multidisciplinary approach not only advances our understanding of animal ecology but also supports law enforcement agencies in wildlife preservation and conservation efforts. Continued research in this field holds promise for expanding the applications of forensic odontology in wildlife conservation and crime investigation. Ultimately, this contributes to the protection of endangered species and the preservation of biodiversity.

## Conclusions

The present study quantified the enamel rod patterns of three different species. The disparities in enamel structure among these mammals could serve as valuable indicators of their evolutionary history, taxonomy, and identification. Despite the limitations of the present study, it can be inferred that each mammal possesses its own distinct enamel rod pattern, and identifying a deceased individual using toothprints may prove challenging if the morphology is not well understood. Therefore, in forensic odontology, a thorough understanding of mammalian enamel morphology is essential for accurate identification, particularly when relying on toothprints to determine the identity of a deceased individual.
